# Expression and Antigenic Evaluation of *VacA* Antigenic Fragment of *Helicobacter Pylori*

**Published:** 2013-07

**Authors:** Leila Hasanzadeh, Ehsanollah Ghaznavi-Rad, Safieh Soufian, Vahideh Farjadi, Hamid Abtahi

**Affiliations:** 1 Department of Biotechnology and Microbiology, School of Medicine, Arak University of Medical Sciences, Arak, Iran; 2 Department of Microbiology and Immunology, School of Medicine, Arak University of Medical Sciences, Arak, Iran; 3 Biology* Department, Payame Noor University, Arak, Iran*; 4 Department of Microbiology, Islamic Azad University, Qom Branch, Qom, Iran; 5 Molecular and Medicine Research Center, Department of Microbiology, School of Medicine, Arak University of Medical Sciences, Arak, Iran

**Keywords:** Antigenic region, Cloning, Epitopes, *Helicobacter pylori*, *VacA* cytotoxin

## Abstract

***Objective(s)*** ***:*** *Helicobacter pylori*, a human specific gastric pathogen is a causative agent of chronic active gastritis. The vacuolating cytotoxin (*VacA*) is an effective virulence factor involved in gastric injury. The aim of this study was to construct a recombinant protein containing antigenic region of *VacA* gene and determine its antigenicity.

***Materials and Methods:*** The antigenic region of *VacA* gene was detected by bioinformatics methods. The polymerase chain reaction method was used to amplify a highly antigenic region of *VacA *gene from chromosomal DNA of *H. pylori*. The eluted product was cloned into the prokaryotic expression vector *pET32a*. The target protein was expressed in the *Escherichia coli BL21 (DE3) pLysS*. The bacteria including *pET32a-VacA* plasmids were induced by IPTG. The antigenicity was finally studied by western blotting using sera of 15* H. pylori* infected patients after purification.

***Results:*** Enzyme digestion analysis, PCR and DNA sequencing results showed that the target gene was inserted correctly into the recombinant vector. The expressed protein was purified successfully via affinity chromatography. Data indicated that antigenic region of *VacA* protein from *Helicobacter pylori* was recognized by all 15 patient’s sera.

***Conclusion*** *:* Our data showed that antigenic region of *VacA* protein can be expressed by in *E. co.li*. This protein was recognized by sera patients suffering from *H. pylori* infection. the recombinant protein has similar epitopes and close antigenic properties to the natural form of this antigen. Recombinant antigenic region of *VacA* protein also seems to be a promising antigen for protective and serologic diagnosis .

## Introduction

*Helicobacter pylori* is a Gram negative curved microaerophilic bacilli colonized in the human gastric mucosa and establishes a chronic infection that is tightly associated with atrophic gastritis, peptic and duodenal ulcers, gastric adenocarcinoma and gastric MALT (Mucosa Associated Lymphoid Tissue) lymphoma (1, 2). It was recently defined as a group 1 carcinogen (3). Moreover, It is estimated that 60% of the world’s population are infected with* H. pylori*, ranging from 20-30% in developed countries up to 70- 80% in developing countries (4, 5). An important virulence factor of *H. pylori *is a secreted toxin known as vacuolating cytotoxin (*VacA*) that induces the formation of intracellular vacuoles in epithelial cells. This protein causes multiple alterations in human cells including cell vacuolization, depolarization of membrane potential, membrane channel formation, alteration of mitochondrial membrane permeability, apoptosis, disruption of endosomal/lysosomal function, activation of mitogen-activated protein kinases, inhibition of antigen presentation, and inhibition of T-cell activation and proliferation (6-9). Recent molecular and cellular studies of *VacA* action have shown that it is a major virulence factor that is involved in the pathogenesis of inflammation in *H. pylori*–induced gastritis and ulceration (10, 11). This protein is a proper candidate for vaccines. In order to stimulate more specific immune response and facilitate the expression of antigen, the aim of this study was to construct a recombinant vector containing antigenic region of *VacA* from *H. pylori* using immunodominant region of this antigen instead of complete sequence and its expression in *Escherichia coli*, as well as determining its antigenicity as a vaccine candidate and usefulness in serologic diagnosis of *H. pylori* in human.

## Materials and Methods

This research is an experimental study and its ethical code from the ethical committee of Arak University of Medical Sciences, Arak, Iran is 90-118-10.

***Bacterial preparation***

*H. pylori *was cultured from biopsy specimens of dyspeptic patients undergoing routine diagnostic endoscopy. Informed consents to participate in the study were obtained from all patients before the biopsy. Culture conditions were as previously reported (12). Briefly, the isolated biopsy was grown on Brucella agar plates (Merck, Germany) containing trimetoprim (5 μg/ml), vancomycin (10 μg/ml), amphotericin B (2.5 μg/ml), supplemented with 5% sheep blood. Following 3-5 days incubation under microaerophilic atmosphere (Co₂ 10%) at 37°C, the grown bacteria were identified as *H. pylori *by routine microbiologic assays including urease, catalase, and oxidase tests and Gram staining. 

***Detection of antigenic region***

Since the most prevalent *VacA *genotype of *H. pylori *among Iranian strains is s1m2 (13), in order to find out antigenic region, the sequence of *VacA* gene with s1m2 genotype (4243 base pair, 1323 amino acids) which encodes the 142.973 Kilodaltons (KDa) protein, from a reference strain (NCBI GenBank, Accession number: U95971, protein id: AAC25911.1) was submitted to ABCpred, Bcepred and Emboss Antigenic web servers (14).

***Chromosomal DNA isolation***

Genomic DNA of *H. pylori* was extracted from the colonies on the Brucella agar plates according to the standard CTAB/NaCl protocol (15). Quality and quantity of the purified genomic DNA was assessed by 0.8% horizontal agarose gel electrophoresis in 1X TBE buffer (10X TBE: 890 mM Tris-base, 890 mM Boric acid, 25 mM EDTA) and visualized by ethidium bromide (1 µg/ml) staining on UV transilluminator, and spectrophotometry (eppendorf) in 260 nm(15).

***Gene amplification***

Two specific PCR primers were designed with Oligo5 software. The gene of *H. pylori VacA* was amplified from the genome of *H. pylori *by PCR method using the primers (5' GGAATTCTATAAAGCTTCTCTTACC 3') as forward primer and (5' CATCTCGAGTGCTTTAATGTCATTG 3') as reverse primer that contained EcoRІ and XhoI sites, respectively.

PCR amplification was performed in a 50 μl total volume containing 3 μl of template DNA, 2 μl of each primers (10 picomole), 8 μl MgCl₂ (25 mM), 1.5 μl dNTP mixture, 5 μl PCR buffer (10X) and 1 μl of expand DNA polymerase (Roche, Germany). The following conditions were used for amplification: initial denaturation at 94°C for 5 min followed by 30 cycles of denaturation at 94°C for 1 min, annealing at 53°C for 1 min and extension at 72°C for 1 min and further extension for 5 min at 72°C (15). The PCR product was analyzed by horizontal agarose gel electrophoresis in 1X TBE buffer and visualized by ethidium bromide staining on UV transilluminator.

***Cloning of VacA gene in bacterial expression vector and transformation***

The PCR product was purified from the agarose gel by high pure PCR product purification kit (Roche, Germany) according to the manufacturer guideline. It was digested with EcoRI and XhoI, and inserted into the expression vector *pET32a (+)* (Novagene, USA) containing N-terminal histidine tag (6His.tag) which was digested by corresponding restriction endonuclease enzymes using T4 DNA ligase enzyme (Cinagen, Iran) at 22°C during 1 hr incubation. It was afterwards identified by restriction enzyme digestion and PCR.

*E. coli DH5α* and *E. coli BL21 (DE3) pLysS* competent cells were prepared by calcium chloride (CaCl₂) method (15) and were used for transformation of plasmid. The plasmid *pET32a-VacA* was transformed into competent *E. coli DH5α* (Stratagene, USA) as the primary host in order for amplification of recombinant plasmid and subsequently into *E. coli BL21 (DE3) pLysS* (Stratagene, USA) cells as the expression host for recombinant protein production using ampicillin and chloramphenicol resistance for selection (15). The sequence of inserted fragment was analyzed by dideoxy chain termination procedure as described by Sanger *et al* (16).

***Gene expression, purification***

The recombinant *E. coli BL21 (DE3) pLysS* was grown in 2 ml nutrient broth medium being supplemented with ampicillin (100 mg/ml) and chloramphenicol (35 mg/ml) on shaking incubator for overnight at 37°C. On the next day, 500 μl of culture was inoculated in 50 ml of Nutrient Broth medium (0.5 g yeast extract, 1 g Bacto pepton, 0.1 g glucose, 0.5 g NaCl, 0.05 g KCl, 0.025 g MgCl₂.6H₂O, 0.025 g CaCl₂, 0.25 g nutrient broth, 14 μl ampicillin, 14 μl chloramphenicol), at 37°C with vigorous agitation at 220 rpm. The cells grew until the OD (Optical Density) at 600 nm reached 0.5-0.8.

Expression of the *VacA* protein was induced by the addition of 50 μl Isopropyl--D-thiogalactopy-ranoside (IPTG) to a final concentration of 1 mM and incubated for four hours. Afterwards, it was harvested by centrifugation at 8000 rpm for 20 min and whole cell lysates before and after induction was examined by sodium dodecyl sulfate polyacrylamide gel electrophoresis (SDS-PAGE) 12%. The expressed protein was purified by affinity chromatography with Ni-NTA agarose resin according to manufacture instruction (Qiagen, USA). In this case, urea was applied and the purified protein was subsequently dialyzed with phosphate buffered saline (PBS) (pH= 7.2) at 4°C overnight. The quality and quantity of purified recombinant immunodominant region of *VacA* protein was analyzed by SDS-PAGE 12% and spectrophotometry (260/280 nm) methods, respectively (15).

***Western Blot analysis***

The integrity of the product was confirmed by western blot analysis. Western blotting was performed according to the standard protocol (15) using sera from a 15 *H. pylori *sero-positive patients (which were detected positive by the urease test, pathological dying and culture) and 15* H. pylori* negative human sera as negative control (which were detected negative by the above mentioned examinations) as primary antibody with 1:100 dilution and HRP-conjugated anti-human IgG (ABcam, United Kingdom) in 1:1000 dilution in 1X TBST buffer (10X: 15 mM NaCl, 10 mM Tris-HCl (pH=7.4), 0.1% Tween 20) as secondary antibody. 

***Statistical analysis***

The statistical analysis of data was performed using SPSS software (version 18: SPSS Inc., Chicago). Also used of chi-square test.

## Results

***Detection of antigenic region***

**Figure 1:**
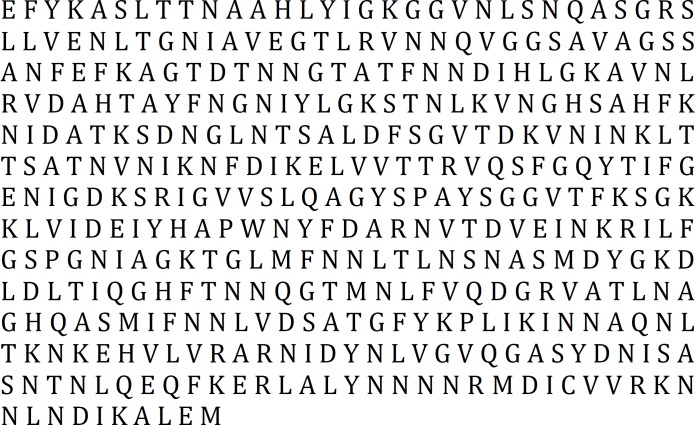
The result of antigenic fragment

The result obtained from the three above-mentioned servers was in accordance; therefore, nucleotide sequence of 1224 till 2457 was selected as region with high antigenic properties (Figure 1).

***Bacterial culture and DNA amplification***

**Figure 2:**
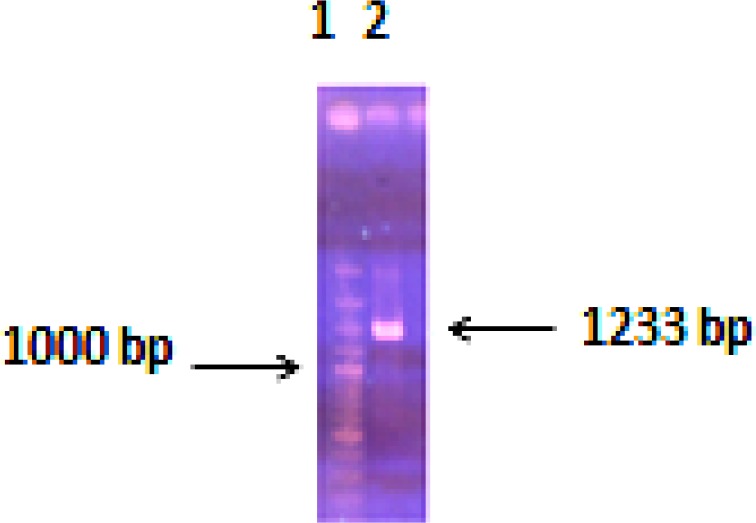
Antigenic region of the *VacA* gene from *Helicobacter* pylori amplified by PCR. Lane 1: DNA marker, Lane 2: PCR product

Genomic DNA of *H. pylori* was extracted from the culture and its concentration was 250μg/ml. The PCR technique was used to amplify the highly antigenic region of the *VacA* gene from H. pylori genomic DNA. The amplified fragment had the expected size of 1233 base pair (bp) comparing to 100 bp DNA ladder (Figure 2). 

***Construction of recombinant plasmid***

the recombinant plasmid was sequenced by Sanger method and the sequencing result was confirmed comparing with databases using basic local alignment search tool (BLAST) software of the NCBI. The results indicated that the target gene was inserted correctly into the vector and has 100% homology with reported original sequence. At the same time, it was successful in transforming recombinant plasmid into *E. coli* strain.

***Expression and purification of recombinant protein***

**Figure 3:**
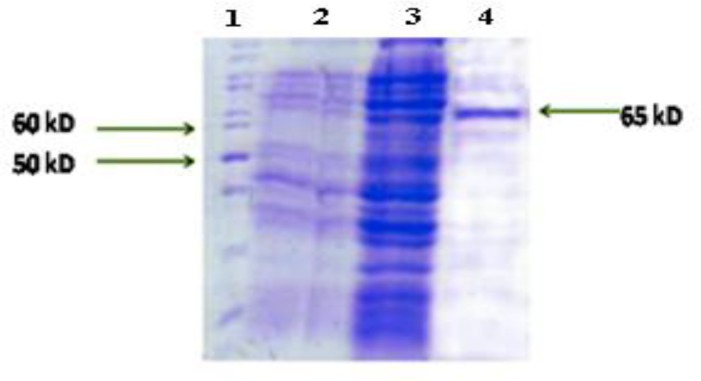
Expression of recombinant antigenic fragment of *VacA* protein and its purification. Lane 1: Protein marker, Lane 2: *pET32a-VacA* before induction, Lanes 3: *pET32a-VacA* after induction, Lane 4: purification of recombinant *VacA* protein.

*pET32a-VacA* was introduced into *E. coli BL21 (DE3) pLysS* and the expression of recombinant protein was induced by IPTG. A band in the range of molecular weight ~65 KDa was found in SDS-PAGE analysis that was similar to what had been predicted (Figure 3). Due to the presence of 6His.tag at the N-terminal region of r*VacA*, purified recombinant protein was obtained using Ni-NTA resin in an affinity chromatography procedure (Figure 3). The concentration of highly expressed recombinant protein was calculated and 2.1 mg/ml was obtained by further purification and dialysis.

**Figure4:**
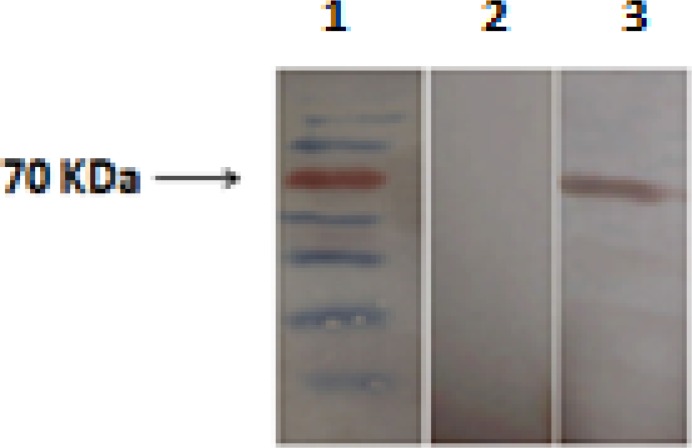
Western blot analysis of recombinant antigenic fragment of *VacA* protein. Lane 1: Protein marker, Lane 2: Western blotting by *Helicobacter pylori* negative sera from patients (negative control), Lane 3: Western blotting by *H. pylori* positive sera from patients

***Immunoblotting analysis***

In order for determining the antigenicity of recombinant antigenic fragment of *VacA *protein in *H. pylori *positive sera of patients, identity confirmation of r*VacA* was applied via western blotting and the desired band was observed with an estimated molecular weight of ~65 KDa. All 15 *H. pylori *infected sera were interacted with recombinant *VacA* protein, whereas all 15* H. pylori* negative human sera failed to act similarly. The specific interaction between patient’s sera and purified r*VacA* protein is shown in Figure 4. The* H. pylori* negative sera of patients were used as negative control.

## Discussion

In the present study, we have shown that recombinant antigenic region of *VacA* protein can be produced by *pET32a (+)* in *E. coli*. Our data indicate that antigenic protein of *VacA* of *H. pylori* could be detected by all 15 patient’s sera; therefore, this recombinant *VacA* can be used for detecting anti-*VacA* antibody.

Currently, the main treatment to eliminate *H. pylori* is combined antibiotics therapy. However, due to incomplete eradication of* H. pylori *through antibiotic treatment, the cost of combination therapy, re-infection, patients’ complaints and emerges of antibiotic resistance strains, vaccination could be suggested as a preventive approach (17). The *VacA* has been approved as an effective virulence factor involved in the pathogenesis of *H. pylori* infection (12, 18, 19). Thus, this protein can be considered as a vaccine candidate.

VacA is initially encodes as a 140-KDa precursor protein that subsequently undergoes both N-terminal and C-terminal processing to yield an 87-95-KDa mature secreted toxin (20, 21). Mature secreted *VacA* toxin yields two fragments that are 33 and 55 KDa (p33 and p55) (22). The N-terminal p33 domain contains a hydrophobic sequence involved in pore formation, and the p55 domain contains cell-binding domains (23).

Mature secreted *VacA* assembles into a large flower-like oligomer in the extracellular space. The oligomer is disassembled by exposure to acid or alkaline conditions. The *VacA* monomer associates with forming anion-selective membrane channels (11). 

The intact protein contains full-length DNA sequence and unneeded epitopes and due to the large size of gene fragment, can result in a higher molecular weight, which makes it difficult to clone, express and purify; hence, limits this protein to be used in whole sequence as a vaccine candidate. In this study, in order to increase the immunogenicity and decrease the ineffective immune response, part of *VacA* gene with suitable antigenic properties, which had been determined with bioinformatics methods, was used for detecting the antibody. Therefore, the protein obtained in our study is smaller than normal *VacA* protein with the same antigenic properties. The yield of highly expressed recombinant antigenic fragment of *VacA* protein was calculated 2.1 mg/ml.

In Talebkhan *et al* study, a full sequence of *VacA* gene (2523 bp) was utilized to produce recombinant protein in *E. coli* and the recombinant protein was obtained with molecular weight of approximately 95 kDa (12).

In Liu *et al* study, three protective proteins *UreB*, *VacA* and *CagA *were chosen as candidate antigens for the vaccine design. In order to facilitate the expression of antigens, some frags (*CagA*: aa 230-389, *VacA*: aa 744-805 and *UreB*: aa 250-387) were chosen to construct a new fusion protein, and live attenuated *Salmonella typhimurium* vector vaccines, which expressed the fused protein arrangement CVU consisting of* CagA*160, *VacA*62 and *UreB*138, could significantly reduce *H. pylori* colonization in infected mice (24). 

A small deletion mutation (targeting aspartic acid 346 and glycine 347) within the p55 amino-terminal subdomain was introduced into the *H. pylori* chromosomal *VacA* gene by Ivie et al. Similar to wild type *VacA*, the *VacA* ∆346-347 mutant protein was proteolytically processed, secreted, and bound to eukaryotic cells. However, *VacA* ∆346-347 did not induce cell vacuolation or membrane depolarization, and its ability to assemble into large water-soluble oligomeric structures was impaired. *VacA* ∆346-347 could physically interact with the wild type of *VacA* to form mixed oligomeric complexes, and inhibited vacuolating activity of the wild type in a dominant-negative manner (25).

In order for giving a high-level expression of recombinant protein, we used *E. coli BL21 (DE3) pLysS* due to cytoplasmic protease deficiency in this strain (26).

Expression of protein in *pET* system added several **amino acid**s such as thioredoxin protein (109 amino acids) (Trx.tag) and 6His.tag and S.Tag sequences to recombinant peptide that is beneficial for detection and purification of target protein through affinity chromatography procedure. These additional **amino acid**s increase the molecular weight from 7900 to 28000 of synthesized peptide (27) that there is no interference in fused amino acids (28). 

In this study, we have cloned and expressed immunodominant region of gene that encodes *VacA* under the control of T7 promoter. Our data shows that recombinant antigenic region of *VacA* can be detected as an antigen by all 15 sera from patients suffering from *H. pylori* infection, although all 15 *H. pylori* negative human sera in the control group failed in this matter. Therefore, recombinant antigenic region of *VacA* protein have same epitopes with natural form of this antigen and might be a promising antigen for serological diagnosis of *H. pylori *infections and as a vaccine component. 

## Conclusion

Data indicates that antigenic region of recombinant *VacA* protein from *H. pylori* were recognized by all human sera infected with *H. pylori*. It can be concluded that antigenic region of *VacA* has an antigenic property that can further be used for development of *H. pylori* vaccine and diagnostic kits.
